# Use of covered stents to treat complex cerebrovascular diseases: Expert consensus

**DOI:** 10.3389/fcvm.2022.934496

**Published:** 2022-09-16

**Authors:** Yueqi Zhu, Huaqiao Tan, Zhongxue Wu, Tielin Li, Lianting Ma, Jianmin Liu, Hongqi Zhang, Yuxiang Gu, Tianxiao Li, Sheng Guan, Xiaodong Xie, Chuhan Jiang, Zhenwei Zhao, Chuanzhi Duan, Jieqing Wan, Xiaolong Zhang, Wenfeng Feng, Xuying He, Haibin Shi, Qiujing Wang, Dong Lin, Qiuping Li, Weixi Jiang, Guohua Mao, Shu Zhong, E. Chen, Huaizhang Shi, Shaohua Ren, Donghai Wang, Yizhi Liu, Zengpin Liu, Jianliang Wu, Feng Wang, Xuebin Hu, Jun Wang, Fan Zhang, Wenfeng Cao, Donghong Yang, Qingrong Zhang, Lei Wang, Binxian Gu, Guangsen Cheng, Yongcheng Zhang, Chun Fang, Minghua Li

**Affiliations:** ^1^Department of Radiology, Shanghai Jiao Tong University Affiliated Sixth People's Hospital, Shanghai, China; ^2^Department of Interventional Radiology, Tongji Hospital Affiliated of Tongji University, Shanghai, China; ^3^Department of Neurointervention Center, Beijing Tiantan Hospital Affiliated of Capital Medical University, Beijing, China; ^4^Department of Neurosurgery, Zhujiang Hospital Affiliated of Southern Medical University, Guangzhou, China; ^5^Department of Neurosurgery, General Hospital of PLA Central Theater Command Neurosurgical Institute of PLA, Wuhan, China; ^6^Neurovascular Center, Changhai Hospital, Naval Medical University, Shanghai, China; ^7^Department of Neurosurgery, Beijing Xuanwu Hospital Affiliated of Capital Medical University, Beijing, China; ^8^Department of Neurosurgery, Huashan Hospital, Fudan University, Shanghai, China; ^9^Department of Interventional Radiology, Zhengzhou University People's Hospital, Henan Provincial People's Hospital, Zhengzhou, China; ^10^Department of Neuro-Interventional Radiology, The First Affiliated Hospital of Zhengzhou University, Zhengzhou, China; ^11^Department of Neurosurgery, West China Hospital of Sichuan University, Chengdu, China; ^12^Department of Neurosurgery, The Second Affiliated Hospital of Xian Medical College, Tangdu Hospital, Xi'an, China; ^13^Department of Neurosurgery, Renji Hospital, Shanghai Jiao Tong University School of Medicine, Shanghai, China; ^14^Department of Interventional Radiology, Huashan Hospital, Fudan University, Shanghai, China; ^15^Department of Neurosurgery, Nanfang Hospital, Southern Medical University, Guangzhou, China; ^16^Department of Interventional Radiology, Jiangsu Province Hospital, Nanjing, China; ^17^Department of Cerebrovascular Surgery, The Third Affiliated Hospital of Sun Yat-sen University, Guangzhou, China; ^18^Department of Neurosurgery, Rui Jin Hospital, School of Medicine, Shanghai Jiao Tong University, Shanghai, China; ^19^Department of Neurosurgery, Zhongshan Hospital, Fudan University (Xiamen Branch), Xiamen, China; ^20^Department of Neurosurgery, Xiangya Hospital, Central South University, Changsha, China; ^21^Department of Neurosurgery, The Second Affiliated Hospital of Nanchang University, Nanchang, China; ^22^Department of Neurosurgery, Guangxi Academy of Medical Science, The People's Hospital of Guangxi Zhuang Autonomous Region, Nanning, China; ^23^Department of Neurosurgery, Zhongshan Hospital, Xiamen University, Xiamen, China; ^24^Department of Neurosurgery, The First Affiliated Hospital of Harbin Medical University, Harbin, China; ^25^Department of Neurosurgery, Shanxi Provincial People's Hospital, Taiyuan, China; ^26^Department of Neurosurgery, Qilu Hospital of Shangdong University, Jinan, China; ^27^Department of Interventional Radiology, The First Affiliated Hospital of Soochow University, Suzhou, China; ^28^Department of Neurology, The Second Hospital of Hebei Medical University, Shijiazhuang, China; ^29^Department of Neurosurgery, The Second Hospital of Hebei Medical University, Shijiazhuang, China; ^30^Department of Interventional Radiology, The First Hospital Affiliated Dalian Medical University, Dalian, China; ^31^Department of Neurosurgery, Wuhan Union Hospital, Tongji Medical College, Huazhong University of Sciences and Technology, Wuhan, China; ^32^Department of Neurology, The First Medical Center of Chinese PLA General Hospital, Beijing, China; ^33^Department of Neurointerventional Radiology, Hainan General Hospital, Hainan Affiliated Hospital of Hainan Medical University, Haikou, China; ^34^Department of Neurology, People's Hospital of Jiangxi Province, Nanchang, China; ^35^Department of Neurosurgery, Daping Hospital, Third Military Medical University, Chongqing, China; ^36^Department of Neurosurgery, Nanjing Drum Tower Hospital, The Affiliated Hospital of Nanjing University Medical School, Nanjing, China; ^37^Department of Neurosurgery, Yichang Central People's Hospital, The First College of Clinical Medical Science, China Three Gorges University, Yichang, China; ^38^Cerebrovascular Diseases Department, Zhuhai Hospital Affiliated With Jinan University, Zhuhai, China; ^39^Department of Neurology, Affiliated Hospital of Jinggangshan University, Ji'an, China

**Keywords:** cerebrovascular disease, covered stent, intraluminal reconstruction treatment, expert consensus, endovascular treatment

## Abstract

The treatment of complex cerebrovascular diseases (CCVDs) at the skull base, such as complex intracranial aneurysms, carotid-cavernous sinus fistulas, and intracranial artery traumatic injuries, is a difficult clinical problem despite advances in endovascular and surgical therapies. Covered stents or stent graft insertion is a new concept for endovascular treatment that focuses on arterial wall defect reconstruction, differing from endovascular lesion embolization or flow diverter therapies. In recent years, covered stents specifically designed for cerebrovascular treatment have been applied in the clinical setting, allowing thousands of patients with CCVDs to undergo intraluminal reconstruction treatment and achieving positive results, even in the era of flow diverters. Since there is no unified reference standard for the application of covered stents for treating CCVDs, it is necessary to further standardize and guide the clinical application of this technique. Thus, we organized authoritative experts in the field of neurointervention in China to write an expert consensus, which aims to summarize the results of covered stent insertion in the treatment of CCVDs and propose suitable standards for its application in the clinical setting. Based on the contents of this consensus, clinicians can use individualized intraluminal reconstruction treatment techniques for patients with CCVDs.

## Introduction

Endovascular therapy has been widely used in the treatment of various hemorrhagic cerebrovascular diseases. However, one class of complex cerebral artery lesions, represented by complex cerebral aneurysms, traumatic carotid cavernous sinus fistulas (CCFs), arterial injuries, dissections, and blister-like aneurysms, indicates a challenge both to endovascular treatment and surgery, and satisfactory treatment goals are not always met. As most complex cerebrovascular diseases (CCVDs) are a kind of parent artery wall defect disease, reconstruction treatment using stent graft repair and flow diversion treatment has overcome the deficiencies of traditional coil embolization to achieve positive outcomes in recent years (1–4).

**Figure 1:**
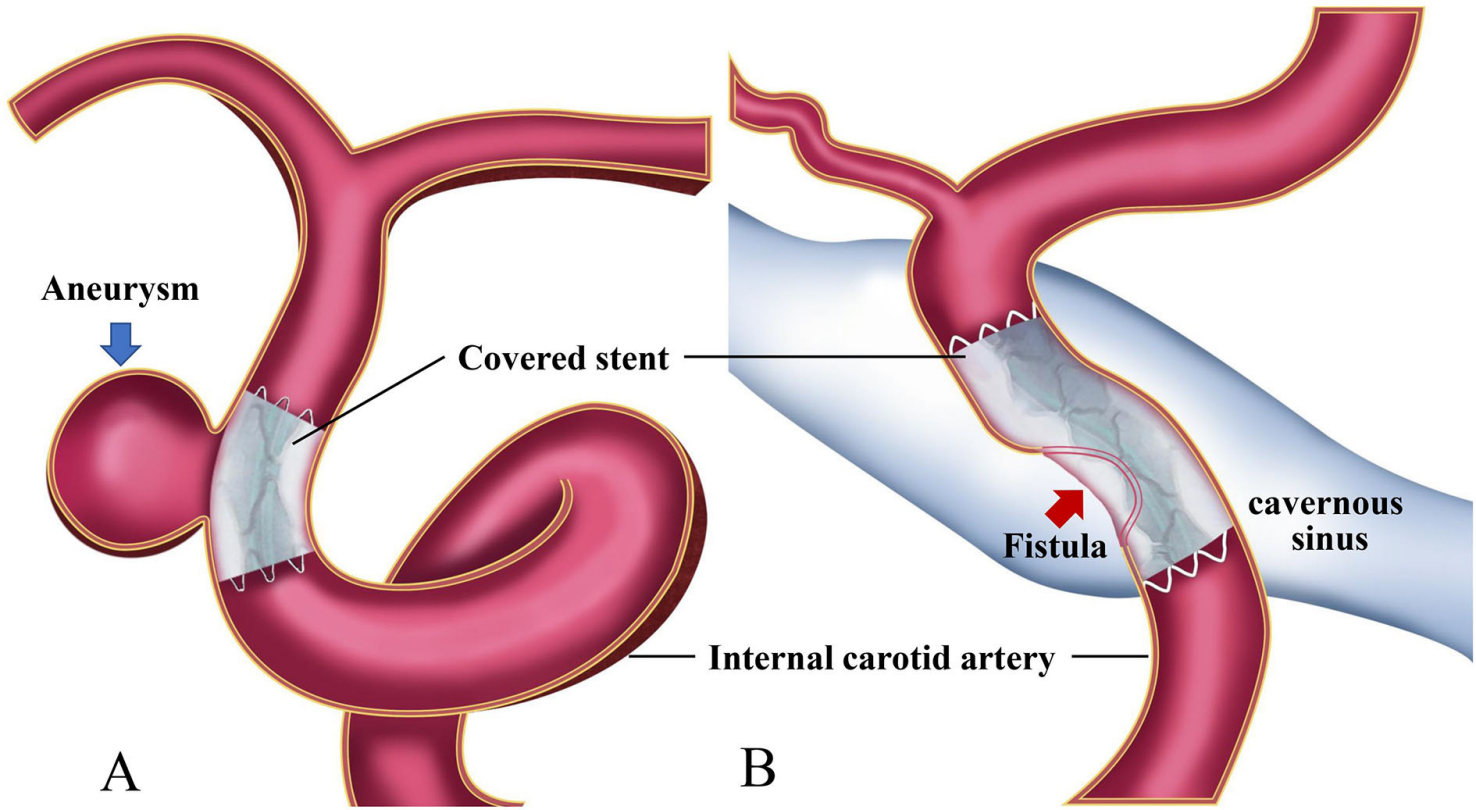
Diagrams of covered stent insertion in the treatment of a cerebral aneurysm **(A)** and a carotid cavernous fistula **(B)**.

Covered stents have been approved and widely applied in China to treat CCVDs at the cervical region or skull base (1, 2, 5–7) (Figure 1). Currently, there are no unified criteria for the application of covered stents for the treatment of CCVDs. Furthermore, unlike more conventional devices, they have some features rendering their use more challenging: difficult delivery, vessel wall injury can occur when deployed in curved vessels, poor membrane adhesion, and high thrombogenicity. The selection of cases and devices, the procedure itself, and the management of complications require standardization to be fully applicable to CCVDs. Consequently, we required authoritative experts with years of experience to review the literature, repeatedly discuss their findings, and form an expert consensus to summarize the current application of covered stents in the treatment of CCVDs and establish a suitable clinical reference standard for their application in clinical settings. Clinical doctors might use the information presented here to tailor individualized endovascular reconstruction therapy regimens for patients with CCVDs according to their specific situation.

## Considerations for intraluminal reconstruction therapy

### Lesion selection

1) Cerebral aneurysms include complex saccular aneurysms, recurrent aneurysms, pseudoaneurysms, dissecting aneurysms, blood blister-like aneurysms, multiple aneurysms at the same site, and so on (8–14).
2) Direct carotid-cavernous fistulas (15–17).
3) Internal carotid artery injuries, including traumatic injuries, iatrogenic injuries, tumor erosions, and so on (18, 19) (Table 1).

No important branches or perforators must be present in the covered artery segment, including the fetal-type posterior communicating artery, anterior choroidal artery, posterior inferior cerebellar artery, anterior spinal artery, and primitive trigeminal artery. For the ophthalmic segment of the internal carotid artery, a balloon occlusion test can be performed to assess the risk of blindness before considering the use of covered stents.

**Table 1:** Comparison of different techniques for endovascular treatment of cerebrovascular diseases.

	**Covered stent insertion**	**Coil embolization**	**Flow diverter deployment**
Indication	1) Complex cerebral aneurysms	1) Most of cerebral aneurysm	1) Unruptured side-wall giant or large cerebral aneurysms
	2) Direct carotid-cavernous fistulas	2) Some kinds of AVMs or fistulas	2) Fusiform or wide-necked aneurysms
	3) Internal carotid artery injuries		
Treatment conception	Diseased parent artery repair	Aneurysm sac occlusion	Blood flow diverting
Target lesion	Parent artery	Aneurysm sac	Impaired flow
Device navigation	Difficult	Easy	Moderate
Technical challenging	Easy for stent deployment	Higher skill required for coiling	Higher skill required for device deployment
Anatomic location	Distal ICA and VA	Most segments as long as the microcatheter can be in place	Distal ICA and VA
Outcomes evaluation	Endoleak type	Reymond classification	OKM scale
Complication	Parent artery injury, in-stent thrombosis, stenosis or occlusion	Aneurysm rupture, parent artery occlusion	Aneurysm rupture, parent artery thrombosis, stenosis or occlusion
Follow up images	DSA, CTA	DSA, MRA	DSA, CTA

AVM, arteriovenous malformation; ICA, internal carotid artery; VA, vertebral artery; DSA, digital substraction angiography; CTA, computerized tomography angiography; MRA, magnetic resonance angiography; OKM scale, O'Kelly-Marotta scale.

### Parent artery selection

Evaluation of the parent artery before receiving covered stent insertion is more important than that of the disease itself. Generally, the parent artery must be relatively straight, including the petrous (C2), lacerum (C3), cavernous (C4), and ophthalmic (C6) segments of the internal carotid artery (ICA), and the V1–4 segments of the vertebral artery (VA), which are selected as the first choice. This requirement can also be selectively applied to other segments according to the specific condition of the patient.

## Key points for the intraluminal reconstruction technique

### Basic considerations for the lesion and its parent artery

**Figure 2:**
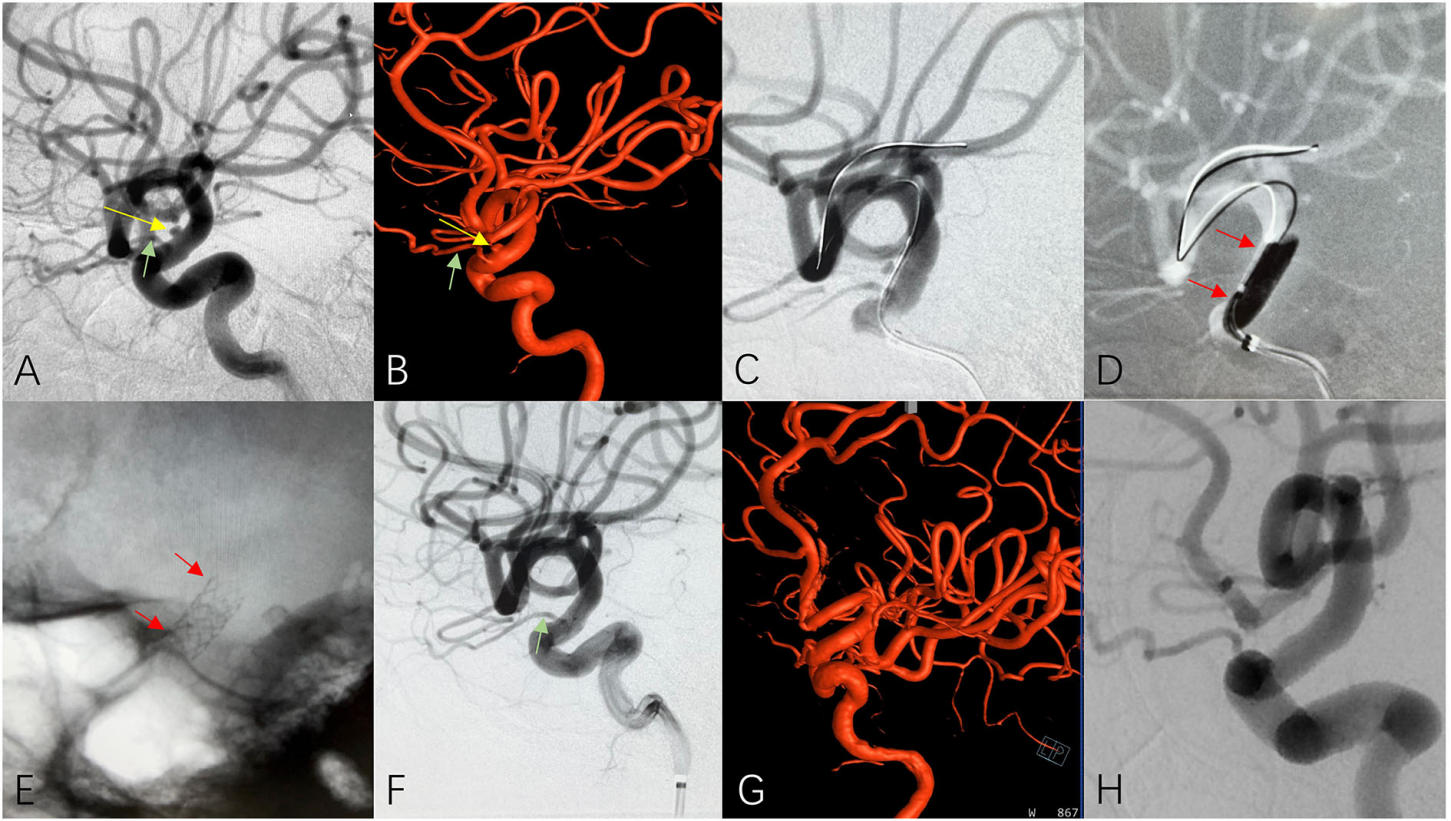
A patient with cerebral aneurysm received covered stent insertion. 2D **(A)** and 3D **(B)** cerebral angiograms reveled a unruptured tiny cerebral aneurysm (yellow arrow) and adjacent ophthalmic artery (green arrow) at the C6 segment of ICA. A covered stent (3.5 × 10 mm) was delivered (**C**, red arrows) released at the diseased parent artery (**D**, red arrows) and the plain film clearly showed well opened covered stent after deployment (**E**, red arrows). Immediate 2D **(F)**, 3D **(G)** and 4-months follow up **(H)** cerebral angiogram confirmed complete disappearance of aneurysm and excellent patency of the ophthalmic artery (green arrow) and parent artery.

Imaging evaluation of the diseased segment artery is important, including the diameter of the proximal and distal parts of the treated segment artery, the length of the lesion in the vessel wall, the tortuosity of the diseased artery, especially the treated segment artery, as well as calcification and plaque in the arterial wall. Arterial wall lesions should generally be <10 mm in length for treatment with a covered stent; if the length of the lesion exceeds 10 mm, overlapping multiple covered stents can be considered if technically feasible and the treatment segment of the parent artery is relatively straight (multiple stents are not recommended for tortuous vessels). If a potentially important perforator is suspected in the diseased segment of the vessel, a high-dose contrast agent is recommended for use in angiography to further confirm its presence. Coverage of any perforating vessels should be avoided to prevent serious adverse consequences (Figure 2).

A relatively straight arterial segment is the ideal condition for the implantation of covered stents. If the artery requiring treatment is not straight, the curvature of tortuosity should not be sharp (generally >130° is recommended) and the lesion is located on the larger side of the curvature of the artery, a covered stent can be considered for treatment. For patients with excessive vascular tortuosity in the segment requiring stenting, stent release by balloon dilation is likely to cause arterial wall damage or intracranial hemorrhage. Similarly, if the lesion is located on the smaller side of the curvature of the tortuous vessel, the implantation of a covered stent may result in endoleak due to its poor membrane adherence to the arterial wall and is thus generally not recommended. The discrepancy between the distal and proximal ends of the treated segment of the artery should not be too large; in principle, if the difference is >0.5 mm, it should be regarded as a contraindication for the application of a covered stent.

### Recommendations for stent size and length selection

It is generally recommended to measure the diameter of the aneurysm sac and parent artery on 2D angiography. Additionally, accurate 3D multiangle measurement is recommended for evaluating the angulation of the parent artery to prevent improper stent selection due to an incorrect measurement or evaluation of the parent artery. The diameter of the covered stent should be essentially the same as or slightly larger (no >0.5 mm) than the vessel diameter of the segment requiring treatment. When patients were in the stage of acute hemorrhage and vasospasm of the target artery was confirmed, the diameter selection of the covered stent should be referred to arterial diameter in its natural status or slightly larger than its currently measured diameter. If the treated artery is located epidurally or interdurally, the choice of diameter for the covered stent can be appropriately enlarged. When the diameters of the distal and proximal ends of the treated segment artery are different, the diameter of the covered stent should be selected according to the diameter of the wide end of the artery segment requiring treatment.

The length of the covered stent should be greater than that of the arterial lesion; generally, the stent should fully cover the diseased arterial wall, including the aneurysm neck, CCF fistula, and wall damage from other causes. A few points are worth noting. First, in the treatment of saccular aneurysms, the length should be 4–6 mm greater than that of the aneurysm neck visible on the best imaging projection. Second, the length of the stent should be chosen as short as possible while guaranteeing the coverage of the lesion in the tortuous vessel, which may prevent vessel wall damage caused by balloon dilation or endoleak caused by poor adherence of the stent membrane to the tortuous artery. Third, the length of the covered stent can be slightly increased if deployed epidurally or interdurally and the segmented artery to be treated is straight. When important branch vessels need to be preserved, if the end of the coated scaffold does not completely cover the branch opening, the undisturbed blood flow of the branch can be preserved in most cases.

### Key technical notes for stent delivery

The stent system consists of a covered stent and a balloon catheter, which makes the whole system stiff runs the risk of failing to arrive at the target lesion, and may cause membrane damage during stent delivery. Therefore, for patients with tortuous pathways, proximal long-sheath support and distal placement of intermediate catheters can be combined to improve the success rate of stent system delivery. For patients with extreme vascular tortuosity, an intermediate catheter with a smaller profile (5 French) and better flexibility is recommended. In rare cases, multiple guidewire support, balloon catheter support, and distal stent deployment support can also be used to assist the intermediate catheter in crossing over the lesion to the distal part of the vessel. By establishing passage with an intermediate catheter, the stent system can be delivered and released smoothly.

### Key technical notes for stent deployment

Negative pressure emptying of the balloon is not recommended until the covered stent is in the correct location. The balloon should be expanded slowly during the deployment of the covered stent to reduce damage to the vessel wall, improve the adhesion between the stent and the vessel wall, and minimize membrane damage. Additionally, attention should be given to controlling the tension of the whole delivery system to reduce the risk of stent migration during balloon expansion. Depending on the degree of vascular curvature, the time from the beginning of balloon dilation to the establishment of nominal pressure should be limited to 1–3 min. When balloon dilatation is sufficient to ensure full opening of the covered stent, it is not necessary to reach the named pressure in the tortuous vessel segment, by which the risk of vessel damage can be minimized. Additionally, slow balloon pressure relief is recommended to avoid poor stent adhesion or stent migration.

### Covered stents and assistant technologies

#### Covered stents and coils

For high-flow CCFs, large/giant aneurysms, or aneurysms located at the curved segment of the vessel, intraluminal covered stent reconstruction treatment has a high risk of leakage due to poor stent adhesion or retrograde filling of the side branches; thus, it is necessary to consider the placement of some coils in the aneurysm sac or within the cavernous sinus before covered stent insertion. For wide-necked aneurysms or high-flow CCFs, the placement of coils in the aneurysm sac or on the distal side of the fistula can support and stabilize the covered stent. Moreover, coiling may be of great help in reducing the flow of the CCF, allowing a clear display of the fistula and ensuring accurate coverage. It is generally recommended to perform coil embolization before deployment of the covered stent. If coil insertion is performed after the deployment of the stent, the risk of internal leakage will increase during coil packing or microcatheter withdrawal. If coil embolization should be performed after stent deployment, the balloon should be placed *in situ*, and rescue dilation should be performed after the microcatheter is removed if necessary.

#### Bare stent-assisted covered stent deployment

This technique is not generally recommended, especially in the era of flow diverters. For some wide-necked large aneurysms involving the parent artery fusiform or dissecting aneurysms, when multiple covered stents are planned to be inserted, the first placed covered stent risks collapsing into the aneurysm sac; thus, a long-bared stent (closed cell stent is preferred) can be placed to provide enough support. These procedures should only be considered for straight vessels, but nevertheless, they are associated with an increased risk of thrombosis or stenosis.

#### Flow diverter-assisted covered stent deployment

The implantation of a covered stent into a flow-diverting device is not recommended as a routine procedure. In a few special cases, if obvious inject flow is observed after immediate flow diverter insertion or the repair of the defect in the wall is not satisfactory after implantation of the flow diverting device after long-term follow-up and other techniques are ineffective, a covered stent can be implanted into the flow diverting device as a rescue method. Similarly, in cases where a non-healing, high-flow endoleak develops after covered stent implantation and rescue coiling or telescoping covered stent insertion is not feasible, a flow diverting device can also be considered as an option for treating the endoleak.

## Management of complications

### Endoleaks

#### Endoleak classification

**Figure 3:**
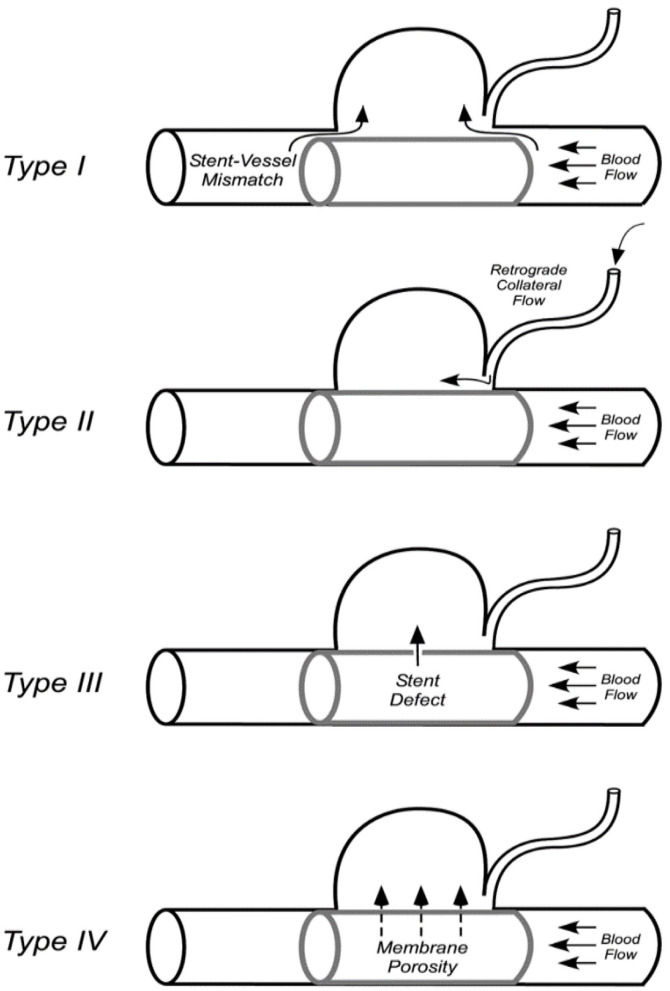
Classification of the covered stent-associated endoleaks (20).

Endoleaks that develop after implantation of covered stent insertion can be classified into four types. Type I: those arising due to poor stent apposition to the vessel wall or stent size mismatch; Type II: retrograde flow filling of the aneurysm sac *via* the collateral vessels; Type III: those arising due to defects of the stent itself; and Type IV: leakage due to membrane pores (Figure 3) (20).

#### General principles for endoleak management

**Figure 4:**
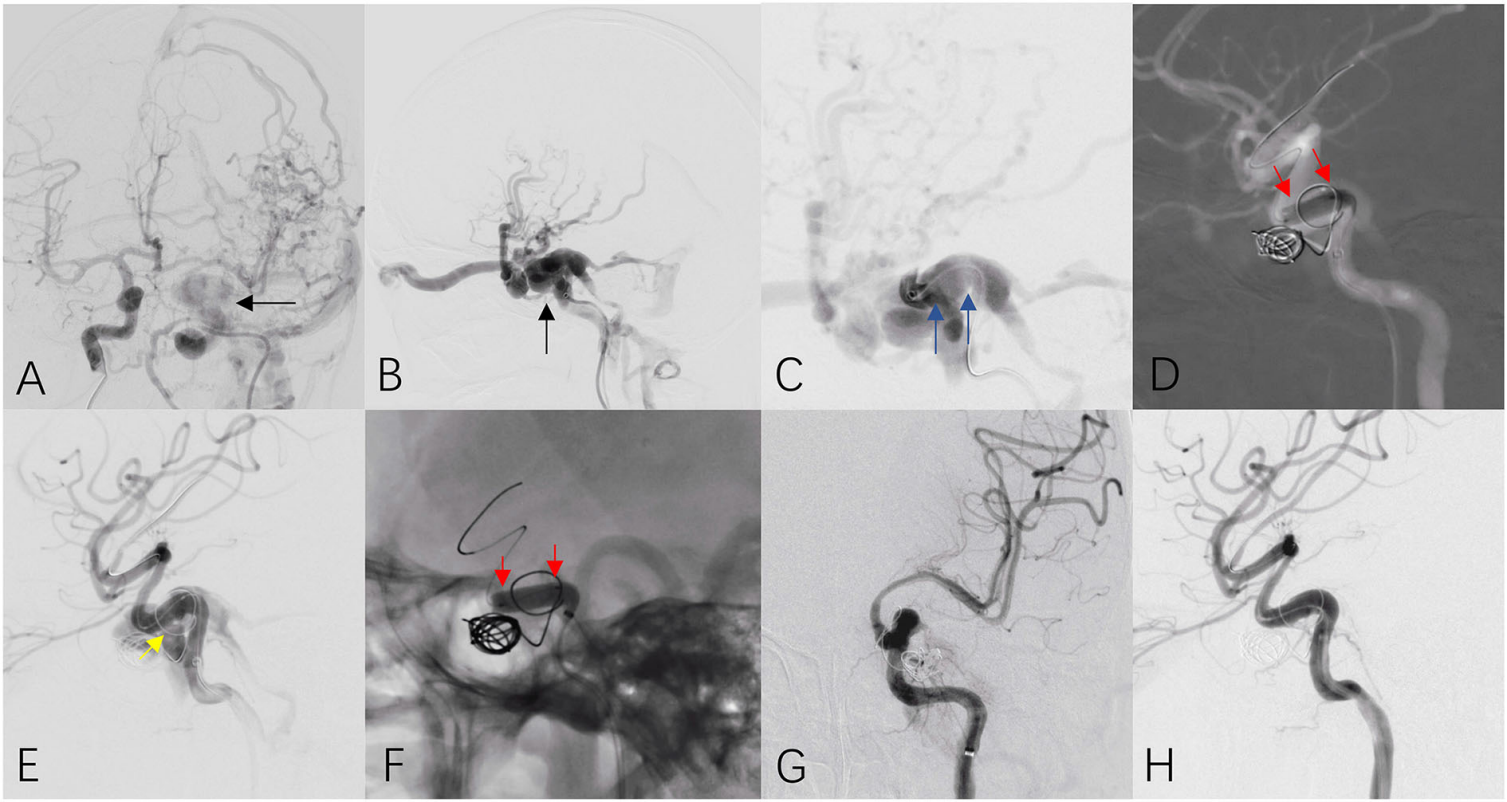
A patient with traumatic carotid cavernous sinus fistula received covered stent insertion. Anteroposterior **(A)** and lateral **(B)** cerebral angiograms revealed high volume of fistula (black arrow) at the C4 segment of the left ICA. Continuous angiogram from an intermediate catheter located the fistula at the horizontal segment at C4 (**C**, blue arrows). A covered stent (4.0 × 13 mm) was released at the diseased parent artery (**D**, red arrows) and immediate angiogram confirmed high volume of type I endoleak at the distal end of stent (**E**, yellow arrow). Post dilation within the stent was performed (**F**, red arrows) and final cerebral angiograms **(G,H)** confirmed complete disappearance of fistula and excellent patency of the parent artery.

i) Selecting the right size for the covered stent (or slightly larger stents released at low balloon pressure) is key to avoiding endoleaks or to determining whether the endoleak can be properly handled. A proximal endoleak in a straight parent artery should be actively managed, while a small amount of distal endoleakage can be observed and followed up (7). For ruptured aneurysms, endoleaks should be actively managed regardless of whether they are proximal or distal (Figure 4).

ii) The balloon should be dilated slowly and the pressure should be increased step by step. For stents that are significantly smaller in diameter than the parent artery, post-balloon dilatation is often ineffective.

iii) For aneurysms located in tortuous artery segments or large or wide-necked aneurysms, some coils should be placed into the aneurysm prior to stenting. Preset microcatheterization may be considered if necessary, or if the leakage is severe, coils or Onyx glue may be used to further occlude the aneurysm.

#### General techniques for treating endoleaks

After covered stent deployment, the balloon should be retained *in situ*. The classification and location of any internal leakage (e.g., distal or proximal end of the stent) should be carefully determined on angiography. If the amount of endoleakage is too large to be distinguished, the balloon can be dilated *in situ* with the same or higher pressure to reduce it. If the endoleak persists, post-dilation should be performed, targeted at the endoleak site by partially moving the balloon forward or backward so that the main body (not the tip or end of the balloon) is located at the endoleak site to increase the adherence of the stent membrane to the vessel wall. If the endoleak is not obvious, multiangle angiography should be performed and then repeated after a 10-min wait before a decision to follow-up observation. It is recommended that the balloon not be expanded more than three times and that the dilation pressure not be too high. When additional covered stent insertion is considered, the size of the stent should generally be the same as or slightly larger than that of the previously inserted stent.

If the above methods are adopted, but the endoleak persists, and there is a high risk of bleeding, occlusion of the parent artery on the affected side or surgical clipping can be considered following sufficient evaluation of the contralateral artery or compensation of the adjacent circulation.

### Vasospasm

After delivery and release of the balloon-dilated covered stent, the target vessels and adjacent branches may spasm, resulting in the illusion of a complete occlusion image. However, after the vasospasm resolves, there is a possibility of endoleak recurrence. Therefore, angiography should be performed again after a period of time (or after the use of antivasospasm drugs).

### Thrombotic events

In addition to a metal scaffold, the covered stent includes an outside membrane. After the covered stent is implanted into the curved segment of the vessel, the membrane can crumple and pile up, especially on the smaller side of the curvature, resulting in thrombosis. Similarly, incomplete opening or poor adherence of the covered stent after implantation at the curved segment can also demonstrate high thrombogenic properties (21).

Adequate pre- and post-operative double antiplatelet therapy, thromboelastography or genetic testing to determine whether there is resistance to drugs, intraoperative heparinization and postoperative anticoagulation are key to preventing thrombotic events after stent implantation. If acute in-stent thrombotic events occur during or after stent implantation, antegrade flow can be restored through the application of platelet GPIIb/IIIa receptor antagonists, balloon dilation, or other mechanical thrombectomy techniques. For some high-risk patients, a target vessel balloon occlusion (BOT) test is recommended before implantation of a covered stent.

### Vessel rupture

Vascular rupture and bleeding caused by the release of covered stents are the most serious complications of their implantation. Improper stent size selection, tortuosity of the treated segment, and sclerosis of the arterial wall are risk factors for rupture of the parent artery. It should be noted that since the covered stent is a balloon-expandable stent, the balloon is longer than the stent, and the flexibility during balloon expansion is poor, arterial wall damage can easily develop in torturous vessels, leading to vessel rupture. Therefore, when choosing the length of the covered stent, it is necessary to consider the potential risk of a longer balloon length during dilation. Remedial measures after hemorrhage mainly include fast balloon re-expansion for hemostasis and occlusion of the parent artery.

### Delayed aneurysm rupture

The main cause of delayed aneurysm rupture is the persistence of various types of endoleaks. Therefore, the main measure to avoid delayed aneurysmal bleeding is to resolve the leakage.

### In-stent stenosis

The incidence of stenosis after covered stent implantation is affected by many factors, such as clinical factors, lumen diameter, vessel curvature, and hemodynamics. The late restenosis rate after covered stent insertion is low. The rates of lumen loss at the site of stent implantation at 2 and 6 years are only 18.0 ± 13.3 and 29.0 ± 18.5%, respectively, compared to those immediately after implantation (1). Stenosis at the site of covered stent implantation is closely related to delayed endothelialization. Studies have shown that covered stent endothelialization can be significantly delayed with respect to that of bare stents and can occur later in curved segments than in straight segments. Complete endothelialization of the covered stent usually takes 6–12 months, so a dual antiplatelet aggregation therapy of at least half a year is recommended (18). Smoking and stent angulation have been identified as risk factors for predicting late in-stent stenosis (1).

### Covered membrane bulging

After the covered stent is implanted, local lumen dilation or bulging can be observed during noninvasive angiography or angiographic follow-up, which usually occurs in the neck of sidewall aneurysms or in the body of fusiform/dissected aneurysms, and it is necessary to identify whether an endoleak has developed. This angiographic finding may be related to the bulging of the local membrane of the covered stent, which is more likely to occur in the body of the covered stent and the parts lacking vessel wall support. A possible mechanism is a loose connection between the coated membrane and the scaffold by suturing; this design is intended to provide a redistribution of the membrane as the stent at the smaller side of the curved segment shrinks. This may be the primary cause of membrane bulging under continuous hemodynamics, a phenomenon that should be harmless and thus recommended for regular clinical follow-up.

## Perioperative medication

### Preoperative antiplatelet aggregation therapy

A combination of dual antiplatelet aggregation agents, clopidogrel (75 mg/day), and aspirin (100 mg/day) is recommended. Oral administration should begin 3–5 days before surgery. Routine preoperative thromboelastography is recommended. If clopidogrel resistance is confirmed, the more potent P2Y12 receptor inhibitor ticagrelor may be substituted; otherwise, the load dose, 300 mg clopidogrel and 300 mg aspirin, should be taken orally or by other means 4 h before surgery.

### Intraoperative heparinization

Intraoperative heparin should be administered *via* intravenous injection with an initial dose of 4,000–5,000 U or 60–80 U/kg for a bolus injection, followed by a half-dose mass injection in the second hour, and so on, with at least 1,000 U added every hour to maintain the patient's systemic heparinized state (activated clotting time (ACT) maintained at more than two times the basal level).

### Postoperative anticoagulation and antiplatelet aggregation therapy

Short-term anticoagulant therapy can be considered after covered stenting. A subcutaneous injection of low-molecular-weight heparin 4,000–5,000 U every 12 h for 3 days is recommended. Oral clopidogrel (75 mg/day) and aspirin (100 mg/day) should be taken for at least 6–12 months; long-term oral administration of a single antiplatelet aggregation agent is then recommended (22).

If an endoleak is obvious in a ruptured aneurysm or there is a high risk of hemorrhage after covered stenting for other lesions, the use of low-molecular-weight heparin can be reduced as appropriate.

For emergency stent implantation, the intraoperative use of platelet GPIIb/IIIa receptor antagonists (e.g., tirofiban) is recommended if unconditionally taken for 3–5 days as a dual antiplatelet aggregation agent before surgery. If postoperative CT confirms no increased hemorrhage, then an overlapping antiplatelet aggregation agent (aspirin 300 mg, clopidogrel 300 mg) can be given. The platelet GPIIb/IIIa receptor antagonist should be discontinued 4–6 h later.

If necessary, hormonal therapy can be used to reduce inflammatory reactions after treatment of a giant aneurysm with a covered stent. The main reason for such inflammatory reactions is an increase in the volume of the aneurysm sac after thrombus formation, resulting in the stimulation of the meninges and nerves and compression of the adjacent brain tissue, leading to the aggravation of patient symptoms. However, their clinical effects need to be further evaluated.

## Follow-up protocol

### Image follow-up protocol

Similar to the treatment of complex cerebrovascular diseases with other technologies, imaging follow-up after covered stent insertion is needed for a short period of 3–6 months, a medium period of 1–2 years, and a long period of more than 5 years. Catheterized angiography is preferred for imaging follow-up. However, the metal struts of the covered stent are thicker than those of intracranial stents and have better radiopacity. Therefore, for cases with covered stent implantation alone, both VR and MIP reconstruction of CTA can clearly show the stent, while multiplane reconstruction and curved reconstruction of thin-layer MIP-CTA, referring to thin-layer coronal images of CTA, can clearly show the patency of the covered stent and isolation of the aneurysm sac. Therefore, CTA can be used as an effective supplementary follow-up method. Magnetic resonance angiography (MRA), including TOF or contrast-enhanced MRA, shows almost complete loss of vascular signal in the stented segment and is therefore not recommended as an imaging follow-up method. In the case of combined coil embolization, catheterized angiography is preferred due to radiative artifacts caused by coils. For cases with sustained endoleak, close imaging follow-up is needed, and whether further intervention is required depends on the location and volume of the endoleak.

### Clinical follow-up protocol

After stent implantation, short-term (1–3 months) and subsequent annual clinical follow-ups are routinely recommended. For cases with endoleak, it is necessary to adjust the use strategy of antiplatelet drugs in time according to the patient's symptoms and follow-up imaging results.

## Economic analysis

By extracting and analyzing direct medical cost data, including the costs of drugs, medical devices, daily hospital beds, nursing, and examinations, a decision tree model was established to evaluate the health costs and efficacy of the treatment of intracranial aneurysms (diameter >7 mm) with covered stents and coil embolization. The results showed that the direct medical costs of covered stent insertion and coil embolization were 21,860.4 and 27,391.7 dollars, respectively, and the aneurysm recurrence rates were 0 and 28.9%, respectively. Therefore, the therapeutic effect of covered stents is better than that of coil embolization, with an incremental cost-effectiveness ratio (ICER) of −9,735,732.7 dollars/death averted. Thus, coated stents can improve the clinical efficacy and reduce the total medical cost for patients with intracranial aneurysms of diameter >7 mm (23).

## Conclusion

Covered stents could become an effective treatment option for complex cerebrovascular diseases, such as complex aneurysms, direct carotid-cavernous fistulas, and internal carotid artery injuries. The requirement for the parent artery for covered stent treatment is higher than that of the disease itself, which indicates that the parent artery must be relatively straight. Endoleak types should be carefully evaluated, and the proximal or high volume endoleaks should be managed properly.

## Author contributions

YZhu: acquisition of data, analysis and interpretation of data and drafting of the manuscript. CF and ML: organize of this expert consensus, critical revision of the manuscript for important intellectual content, and supervision. HT, ZW, TieL, LM, JL, HZ, YG, TiaL, SG, XX, CJ, ZZ, CD, JWan, XZ, WF, XHe, HaS, QW, DL, QL, WJ, GM, SZ, EC, HuS, SR, DW, YL, ZL, JWu, FW, XHu, JWang, FZ, WC, DY, QZ, LW, BG, GC, and YZha: revision of the manuscript. All authors contributed to the article and approved the submitted version.

## Conflict of interest

The authors declare that the research was conducted in the absence of any commercial or financial relationships that could be construed as a potential conflict of interest.

## Publisher's note

All claims expressed in this article are solely those of the authors and do not necessarily represent those of their affiliated organizations, or those of the publisher, the editors and the reviewers. Any product that may be evaluated in this article, or claim that may be made by its manufacturer, is not guaranteed or endorsed by the publisher.
